# Mental profile mapping: A psychological single-candidate authorship attribution method

**DOI:** 10.1371/journal.pone.0200588

**Published:** 2018-07-12

**Authors:** Ryan L. Boyd

**Affiliations:** Department of Psychology, The University of Texas at Austin, Austin, Texas, United States of America; The University of Memphis, UNITED STATES

## Abstract

Modern authorship attribution methods are often comprised of powerful yet opaque machine learning algorithms. While much of this work lends itself to concrete outcomes in the form of probability scores, advanced approaches typically preclude deeper insights in the form of psychological interpretation. Additionally, few attribution methods exist for single-candidate authorship problems, most of which require large amounts of supplemental data to perform and none of which rely upon explicitly psychological measures. The current study introduces *Mental Profile Mapping*, a new authorship attribution technique for single-candidate authorship questions that is founded on previous scientific research pertaining to the nature of language and psychology. In the current study, baseline expectations for results and performance are set using an advanced technique known as “unmasking” on the test case of Aphra Behn, a 17^th^ century English playwright. Following this, Mental Profile Mapping is introduced and tested for its psychometric properties, tested using a “bogus insertion” method, and then applied to canonical Aphra Behn plays. Results from both attribution methods suggest that 2 of 5 questioned plays are likely to have been authored by Behn, with the remaining 3 plays exhibiting a poor fit for Behn’s psychological fingerprint. Mental Profile Mapping results are then decomposed into deeper psychological interpretation, a quality unique to this new method.

## Introduction

Authorship attribution is, broadly speaking, the process by which works of unknown or disputed origins are investigated to determine their history. In the past, various approaches have been adopted to establish authorship information about questioned documents, including methods like the chemical analysis of physical documents, identifying idiosyncratic spellings or phrases (i.e., “stylometry”), and even the formation of subjective, holistic impressions of the contents of a text regarding their fit with a specific authorial candidate (e.g., “this just *feels* like Shakespeare”). Regardless of the specific methodologies, all authorship attribution tasks are inherently forensic in nature: by establishing patterns common to a known author or entity, it is hoped that the general history and origin of texts with unknown authorship can be partially, if not fully, reconstructed. The past 2 decades have seen an explosion of new methods in the world of authorship attribution, particularly those that employ the statistical modeling of language to determine authorship likelihoods [1].

Despite the recent boom in sophisticated text analytic authorship attribution methods, however, tensions often exist in forensic settings where impenetrable algorithms are given free reign over authorship questions to the exclusion of intuitive, digestible, and human insights [2]. Many people tend to have an “algorithm aversion”, or a distrust of opaque algorithms that cannot be easily interpreted by laypersons [3,4]. In simple terms, most people often find it difficult to place blind trust in cold, opaque probability scores generated by a computer, especially when the processes by which such results are generated are poorly understood. Skepticism may be particularly pronounced when it is important for individuals to develop an intuitive understanding of forensic methods and their results [5]. Complex machine-learning methods may not only jeopardize a layperson’s ability to interpret the results of forensic text analyses, but also the ability of expert researchers themselves to adequately understand the process by which results are attained (e.g., by interpreting an algorithm’s resultant model).

In many settings, then, it may be necessary to strike a compromise between sophisticated analytic techniques and deeper, actionable insights that lend themselves to meaningful interpretation. In the case of authorship attribution tasks, this can take the form of methods that create information extending beyond probability statements, such as verifiable idiographic data about an author. For example, rather than a result reading something like “Thomas is 85% likely to be the author of this document”, a more balanced analytic approach should provide additional information in the form of “The author of this document also appears to be impulsive, authoritative, and extraverted, which matches observer reports of Thomas’s personality”.

Notably, methods in the realm of psychological text analysis have advanced by leaps and bounds separate from, yet parallel to, the proliferation of authorship attribution methods. Much recent work in the psychological sciences has found that the psychological properties of an author can be accurately captured using automated text analysis procedures. Research spanning hundreds of labs around the globe have repeatedly found that various categories of language are direct reflections of personality-relevant psychological processes [6,7], suggesting that a person’s mental life can be adequately modeled from even modest language samples [8–10]. In other words, various dimensions of a person’s mental world, such as their emotional, social, and cognitive tendencies, can be captured indirectly, yet accurately, by measuring psychologically relevant patterns in language. Moreover, given the trait-like properties of psychological measures of language [11], it has been found that individuals are uniquely identifiable by the very psychological traces in their language [12].

Such discoveries are paving the way for new combinations of computer science and psychology in a forensic space, however, computational approaches to psychological forensics are currently in their infancy. Many methodological gaps still exist for common tasks such as authorship attribution within each field separately, and virtually no methods exist that successfully combine the two fields to resolve these problem areas. Simply put, most authorship attribution methods are either wholly computational or wholly psychological in nature; these two fields seldom cross paths, yet have great potential for mutual benefit.

The current study brings together computational forensics with psychological forensics by introducing a new method for single-candidate authorship attribution, named *Mental Profile Mapping*, which aims to fill several critical methodological gaps. By combining these two disparate fields into a unified approach, critical gaps are filled within each field, as well as in the broader authorship attribution literature.

### Contemporary authorship attribution methods: Background and gaps

The majority of modern authorship attribution methods use statistical analyses that fall under the umbrella of *supervised machine learning* (SML). SML methods allow a computer system to be “trained” on data where concrete outcomes are known. In practice, trained models can then be used to predict outcomes in new, previously unseen data. For example, if a SML algorithm is trained on a collection of images with known faces, it can be used to accurately identify familiar faces in a novel image as a function of what it has previously learned [13].

The power of SML methods rests in their ability to discriminate between multiple known outcomes with extraordinary accuracy. The appeal of SML in authorship attribution tasks is readily apparent: if a system can be trained on the language patterns to differentiate various known authors, a work of questionable authorship can be statistically assigned to one of those authors with high confidence. These types of problems are known as “multiple-candidate” or “closed-class” problems in authorship attribution–that is, a work is known (or strongly believed) to originate from one of *N* specific authorial candidates. For additional discussion and clarification on the idea of multiple authorial candidates, please see Section A in S1 File.

A considerably more difficult problem in authorship attribution is one of “single-candidate” attribution tasks. In single-candidate problems, the authorship question boils down to “did *Person X* write this text?”. Currently, several methods have been proposed to address single-candidate problems, however, these methods typically operate by converting a single-candidate question into one of multiple candidates. This is often done, for example, by introducing “impostors” who are known *a priori* to have not written the work in question [14]. These methods are useful when comparable data is readily available/accessible in large quantities, however, they are less practical in cases where data is limited or of a unique variety. From a psychological perspective, such methods are also lacking in their ability to furnish deeper insights. Like other authorship attribution methods, most single-candidate attribution methods typically rely on word distributions that provide no further information about an author beyond opaque probability distributions.

To illustrate this last point, consider a hypothetical scenario wherein an unknown author has left behind an unsigned admission of guilt for arson. The police suspect Joseph to be the author of the arson note, and they have obtained several other of Joseph’s “baseline” writings. The question, then, is whether it can be determined that the person who authored the baseline writings (i.e., Joseph) also wrote the admission of guilt. A standard authorship attribution analysis would decay all texts into a series of high-dimensional vectors based on words and word properties (e.g., part of speech, repetition, etc.), then statistically determine the likelihood that they came from the same source using classification algorithms. Particularly when conducting complex tasks such as authorship attribution, advanced statistical and machine learning methods typically preclude any psychological understanding of the person who authored a text [7], which may be of great importance in forensic and courtroom settings.

Now imagine the same scenario described above, yet an analyst employs a *psychological* approach to authorship attribution rather than a purely statistical approach. Using psychological text analysis procedures, an analyst would be able to extract information about Joseph from his baseline texts (e.g., “Joseph’s baseline texts are indicative of someone who is generally extroverted, honest, and unanalytical.”). Furthermore, Joseph’s friends, neighbors, and family also agree that these traits are an apt description of his general personality. In this scenario, the statistical information of standard authorship attribution analyses can be combined with the additional psychological information (e.g., the observer reports of Joseph, the psychological patterns embedded Joseph’s texts) to form a more robust analysis and interpretation of the findings. If the psychological patterns extracted from the admission of guilt are indicative of someone with the same general psychological traits as Joseph, then the forensic account of the arson admission is strengthened.

The above example is a conceptual demonstration of why it is useful, then, to strike a compromise between computational and psychological methods of authorship attribution that satisfies 2 criteria:

1. Valid authorship attribution frameworks must be underpinned by empirical, reproducible, and quantifiable *methods* rather than intuition or subjective judgments (computational/statistical perspective).
2. Ideally, an authorship attribution analysis should provide *results* that can be interpreted in the context of relevant psychological information such observer reports of an individual’s traits, behaviors, and mental profile (psychological perspective).

Such a compromise allows the rigor of advanced authorship attribution methods to be paired with scientifically-validated psychometrics that lend themselves to a deeper, meaningful interpretation from a psychological perspective.

### Modern psychological authorship attribution

Recent research has found that individuals can be differentiated based on their unique psychological composition, which can be reliably measured through language use. Boyd and Pennebaker [12] found strong support for this idea by demonstrating that authors could be robustly and reliably differentiated with near-100% accuracy using exclusively psychological measures of language. Unlike most authorship attribution techniques, which are often based on more atomic linguistic measures (e.g., distributions of short phrases, spelling errors, etc.), Boyd and Pennebaker’s methods lent themselves to a scientific, psychological analysis of an author by identifying their unique psychological attributes.

The ability to engage in follow-up interpretations of language patterns stands in stark contrast to most authorship attribution methods. Furthermore, their results were also able to be compared with observer reports of authors’ personalities, dispositions, and even behavioral events. In their work, Boyd and Pennebaker’s language-based analysis of authors’ psychological profiles showed strong convergence with other psychological data, strengthening the forensic account provided by their authorship attribution analysis. Lewis Theobald, for example, exhibited language patterns consistent with a highly analytic yet socially cold and confrontational personality. Indeed, observer reports of Theobald mirrored the insights gained from language analyses, as he was known to be brilliant but quarrelsome with colleagues and had few to no close friends.

Nevertheless, the methods used in Boyd and Pennebaker’s study of psychological authorship attribution possess some drawbacks that are shared with non-psychological methods. For example, the methods used in their work are only useful for multiple-candidate attribution tasks–single-candidate problems are not typically able to be solved by means of classification tasks alone. Therefore, while Boyd and Pennebaker’s analysis represents a promising first step towards the unification of computational and psychological sciences in the domain of forensic analyses, more work is required to extend and expand this new type of hybrid analysis into uncharted territory.

### Current study

In the current study, a new method of authorship attribution and, more broadly, psychological profiling is introduced. This new method, named *Mental Profile Mapping*, aims to address multiple critical gaps in the forensic space of authorship attribution. Namely, there currently exists a marked lack of empirical, theory-based psychological methods for single-candidate authorship questions. Currently, no explicitly quantitative methods for single-candidate authorship attribution exist in the field of psychology. The methods underlying Mental Profile Mapping not only rely exclusively on psychological features that can be measured from language, but the results of this method can be interpreted in a psychological manner through subsequent decomposition and analysis, thereby providing several of the benefits mentioned earlier.

The current study first demonstrates the results of a high-power and validated authorship attribution method hailing from computer science, known as “unmasking”, which is applied to a test case–the works of Aphra Behn. After baseline results are established in the Behn authorship problem space, Mental Profile Mapping methods are introduced. Results from the new analytic approach are then compared to the unmasking results and explored in the context of other psychological information.

## Methods

### Methodological test case: Authorship attribution with the works of Aphra Behn

For the current study, the plays of Aphra Behn were used as the focal data source for analysis. Aphra Behn is regarded as one of England’s first female playwrights and among the 17^th^ century’s most influential dramatists. Due to the controversial nature of Behn’s writing, as well as gender politics of her own and subsequent periods, much of her work and legacy was suppressed for a considerable stretch of literary history between her death and the latter half of the 20^th^ century [15]. However, Behn’s legacy has rapidly become the object of much study and interest in modern literary scholarship [16].

Born in 1640, shortly after William Shakespeare’s death, Behn’s childhood is shrouded in mystery, and much of her early life history appears to be obscured either intentionally or due to extraneous factors [17]. Behn served as a spy during the Second Anglo-Dutch War prior to becoming a skilled and well-performed playwright, and she is known to have served several tours during wartime [18]. Much of Behn’s literary work was highly successful during its time, and Behn’s death in 1689 caused an upsurge in public demand for published copies of her work.

Profiteers and publishers of the time met the demand for copies of Behn’s work by compiling her plays and commissioning several rounds of printing. In particular, Charles Gildon and Gerald Langbaine helped to fuel additional demand through their (perhaps sensationalized) biographical accounts, occasionally mixed with praise of her personality and exploits. In recent years, doubts have been cast regarding the true origin of some of Behn’s posthumously-published plays, particularly those in which Gildon was involved. Gildon was an occasional literary forger and writer, and his involvement in the publication and dissemination of Behn’s works has raised suspicions about whether the posthumous publications are authentic, or merely opportunistic forgeries [15].

The works of Aphra Behn are a suitable test case for authorship attribution methodologies for several reasons. First, Behn’s plays are composed of tens of thousands of words, which makes them suitable for extracting stable language patterns within each work. Additionally, lengthy writings such as Behn’s plays allow for highly reliable psychological measurements to be performed via automated text analysis [7,19].

Furthermore, it is helpful that plays by several other playwrights surrounding Behn’s era, both before and after her life, are readily available in machine-readable format and exist in the public domain, providing accessible comparison cases for testing. Lastly, scholars have painstakingly assembled thorough timelines of events, records, and other chronological information for the life and works of Behn [18]. Importantly, this last factor allows the results of a psychological authorship attribution analysis to be tentatively compared to details of Behn’s life, facilitating a convergence of empirical methodology with external psychological information.

### Setting an authorship expectation baseline: The “unmasking” analysis

Before introducing the Mental Profile Mapping approach, it is important to first establish basic results within the Aphra Behn authorship problem space using an established, high-powered, and validated technique. By setting baseline expectations, the Mental Profile Mapping analysis can be more thoroughly evaluated for convergent validity. Essentially, if results from the new method are comparable to that of an established computational method, it becomes easier to place faith in the results of both analytic tactics. For the current analysis, a powerful modern authorship attribution technique from the computational sciences was selected to set a baseline for expectations pertaining to authorship results–this method is known as “unmasking” [20].

#### A brief description of unmasking

The unmasking method, like many other authorship attribution methods, requires several texts by multiple known authors to determine the origins of a questioned work. However, unlike similar methods that convert single-candidate problems into multiple-candidate problems, the unmasking method occupies a unique hybrid space between the two types of problems. In essence, unmasking operates by using machine learning methods to model the “depth of difference” between an author’s known works and texts written by other authors. Subsequent unmasking stages then use this “depth of difference” information to classify unknown texts for authorship. Results from the unmasking method come in a fairly straight-forward form: a questioned work either is or is not a match with a given author (with a given probability). Because of the specific process by which unmasking operates, it is a uniquely “open-class” approach to authorship attribution that is suitable for the current comparison.

The unmasking method involves several stages that coalesce into a meta-learning algorithm designed for authorship attribution. The logic of the unmasking method is both quite clever and conceptually simple, yet rather complex in its execution. The underlying idea behind the method is this: if we select one work at random out of an author’s complete works, it would be rather easy to use machine learning methods to differentiate the selected work from the rest of the author’s writings. However, if we were to iteratively remove a handful of those features that best differentiate the selected work from the others by the same author, the differentiation process becomes increasingly difficult with each iteration.

For example, *The Tommyknockers* by Stephen King may have “superficial” differences from his other works, such as different characters and themes, but there will also be several linguistic patterns that are constant throughout his works by virtue of the fact that King himself has created the prose. As one gradually strips away these superficial features (e.g., themes, settings, characters), all of his works become increasingly difficult to linguistically differentiate–this iterative drop in accuracy creates a “prediction degradation curve”. In contrast, if the same process is performed to compare *The Tommyknockers* with the works of several other authors, King’s novel will still be able to be uniquely identified rather easily. Even after the removal of superficial differences between *The Tommyknockers* and works by other authors, King’s imprint on the book’s language remains distinct from other peoples’ writings. In other words, the “fingerprint” of King’s language is ultimately still unique enough to differentiate his work from novels written by other people–there is very little prediction degradation, resulting in a rather shallow (or even flat) curve.

The essence of the unmasking method lies in the generation of these prediction degradation curves. The degradation curves, with some additional information, are used in a meta-learning algorithm to identify when works do (or do not) belong to any given author. In practice, unmasking uses support vector machine (SVM) models with linear kernels. Texts by each author in a corpus are initially tested to see how well they fit into an author’s own corpus versus an amalgam of “different author” works. Initially, results are typically very strong–works by a given author share enough linguistic features to stand apart from the works of all authors.

The basic unmasking process is repeated several times (e.g., *f* = 10). However, during each iteration, a select number of features (e.g., *k* = 2) that best differentiate each work from either the same author or those of others are removed, thus negatively impacting SVM classification accuracy. The reasoning behind this approach is that prediction degradation will occur much more quickly for same-author works than different-author works. The results of each iteration (i.e., “fold”, or *f*) are stored and later used in a separate SVM model (i.e., the meta-learning model) that can identify whether questioned works belong to a given author as a function of their prediction degradation curves.

### Data

#### Aphra Behn corpus

A collection of works by Aphra Behn was provided by Melanie Evans, Elaine Hobby, and Claire Bowditch [21,22] as part of an upcoming compilation of Behn’s works. All texts were provided with modernized spellings and had extraneous text (e.g., dramatis personae, title pages, prefaces) removed. A complete list of Behn’s canonical plays included in the current study is presented in Table 1.

**Table 1:** Aphra Behn plays included in the current analyses.

The Forc'd Marriage	The Rover, Part I*	The Roundheads
The Amorous Prince	Sir Patient Fancy	The False Count
The Dutch Lover	The Feigned Courtesans	The Young King
Abdelazer*	The Rover, Part II*	The Emperor of the Moon
The Town Fop	The City Heiress	The Lucky Chance
The Widow Ranter		

Note: Adaptions of other people’s work are denoted with an asterisk (*).

#### Works of questioned authorship

In addition to the verified works of Aphra Behn, 5 plays of questioned authenticity were provided: *The Debauchee*, *The Woman Turned Bully*, *The Counterfeit Bridegroom*, *The Revenge*, and *The Younger Brother*. All works of questioned authorship were prepared/cleaned in a manner analogous to that described above.

These 5 plays were selected as works of potential Behn authorship along several criteria. First, most of these plays were published between 1675 and 1680, a period during which Behn was at her most prolific. While some of the questioned plays do not bear Behn’s name as an author, most of them are thematically congruent with her known authored works. Two of these plays (*The Debauchee*, *The Revenge*) were marked as possible Behn works due to their use of prostitutes as central characters, whereas *The Woman Turned Bully* and *The Counterfeit Bridesgroom* feature women cross-dressing as men to achieve financial independence–both tropes being markedly common in the works of Behn.

Finally, the last questioned play (*The Younger Brother*) was posthumously published in 1696. While this play was, in fact, attributed to Behn at the time of its publication, it was prefaced by Charles Gildon, who claimed to have made his own edits to Behn’s original work and is commonly viewed as an unreliable source. Given that Behn’s works were a profitable commodity at the time of its publication, this final play can be seen as suspect for true Behn authorship. For additional discussions on the matters of authorship details surrounding these works, please refer to Spencer [15] and O’Donnell [18].

#### Supplemental unmasking corpus: Preparation and analysis

Works by several additional playwrights were collected into a separate corpus as a part of the “unmasking” analysis procedures. Supplemental playwright data contained within the “unmasking” corpus included the works of William Shakespeare (35 plays), Christopher Marlowe (5 plays), John Fletcher (9 plays), Lewis Theobald (12 plays), William Rowley (1 play), and Thomas Dekker (13 plays). Works by these additional authors were selected based on several criteria, primarily their ready availability, as well as their genre similarity to the plays of Behn, and the fact that all supplemental playwrights lived in England within a century of Behn (years of birth and death range from 1564 to 1744; Behn lived from ~1640 to 1689). All supplemental works were gathered independently but were prepared in a manner similar to the works of Behn by means of spelling modernization, extraneous information removal, and so on [23].

### Text analysis method

All plays by all authors were analyzed using the 2015 version of the Linguistic Inquiry and Word Count software (LIWC2015) [24]. The LIWC2015 software codes texts for words belonging to ~80 psychological dimensions of language previously found to be related to emotions, social and cognitive processes, and attentional processes. The LIWC2015 application operates by calculating the frequency of words belonging to each category in a given text, then dividing by the total number of words. For example, if a text has 1 positive emotion word (e.g., “pleasure”) out of 10 total words, the text is scored as 10% for positive emotion words. This method has been extensively validated in research the past 2 decades, and the LIWC categories are often used in authorship attribution studies [25,26]. LIWC features have also been found to be as useful in psychological analyses of authorship attribution as standard n-gram distributions [12]. Additional information on LIWC as a method, as well as how it functions in the context of literary works, is presented in the Sections B and C of S1 File.

Plays by all authors were segmented into chunks of ~250 words and analyzed using LIWC2015 in accordance with unmasking procedures. The unmasking procedure was then applied to the resulting 8,066 play segments in the precise manner outlined in Koppel, Schler, and Bonchek-Dokow [20]. This method was performed using 10 folds in conjunction with a *k* parameter set to 2 (i.e., 4 features dropped per iteration). Additional prediction degradation curve data was included in the overall feature set, including linear and quadratic polynomial beta weights and *i*+*f*_{1, 2}_ degradation Δ scores.

It is important to note that, unlike the majority of other authorship attribution methods, the specific authors selected for unmasking analyses are not of particular importance. Ultimately, the unmasking approach does not cleanly distill into a traditional multiple-candidate authorship problem that must select one of the candidates as the “true” author (i.e., “Text X *must* have been written by one of these authors–therefore, which author most likely wrote Text X?”). Instead, supplemental authors are primarily used as a sounding board for the purpose of modeling same- versus different-author prediction degradation curves.

## Results and discussion

The unmasking method performed extremely well with the psychological data generated by LIWC for each author. The meta-learning algorithm showed strong performance for correctly identifying same-author versus different-author degradation curves across the entire dataset (accuracy = 93.96%; kappa = .74). Results were equally strong when considering same-author versus different-author curves for Behn alone (class-balanced accuracy = 88.89%; kappa = .78).

Ultimately, the goal of the unmasking method is to determine whether works of unknown origins were created by a known author. Typically, the unmasking procedures are useful for considering a single author candidate (e.g., “Did Aphra Behn write *X*?”). However, the results of this method can be looked at more broadly as well, ensuring that other authors who are known to not be “true” candidates are also ruled out. Full results from the unmasking analysis are presented in Table 2.

**Table 2:** Results from the unmasking analysis.

Questioned Play Title	Comparison Author	Authorship Match	Result *p*
The Counterfeit Bridegroom	Behn	Different	82.08%
The Counterfeit Bridegroom	Marlowe	Different	99.83%
The Counterfeit Bridegroom	Fletcher	Different	99.40%
The Counterfeit Bridegroom	Rowley	Different	98.99%
The Counterfeit Bridegroom	Theobald	Different	99.97%
The Counterfeit Bridegroom	Dekker	Different	98.61%
The Counterfeit Bridegroom	Shakespeare	Different	96.05%
The Debauchee	Behn	Different	94.41%
The Debauchee	Marlowe	Different	99.98%
The Debauchee	Fletcher	Different	95.07%
The Debauchee	Rowley	Different	99.71%
The Debauchee	Theobald	Different	100.00%
The Debauchee	Dekker	Different	99.52%
The Debauchee	Shakespeare	Different	98.32%
The Revenge	Behn	Match	99.98%
The Revenge	Marlowe	Different	99.72%
The Revenge	Fletcher	Different	98.10%
The Revenge	Rowley	Different	99.48%
The Revenge	Theobald	Different	99.89%
The Revenge	Dekker	Different	94.90%
The Revenge	Shakespeare	Different	96.88%
The Woman Turned Bully	Behn	Different	88.53%
The Woman Turned Bully	Marlowe	Different	99.93%
The Woman Turned Bully	Fletcher	Different	97.80%
The Woman Turned Bully	Rowley	Different	99.87%
The Woman Turned Bully	Theobald	Different	99.94%
The Woman Turned Bully	Dekker	Different	99.94%
The Woman Turned Bully	Shakespeare	Different	87.51%
The Younger Brother	Behn	Match	55.10%
The Younger Brother	Marlowe	Different	99.93%
The Younger Brother	Fletcher	Different	99.91%
The Younger Brother	Rowley	Different	99.58%
The Younger Brother	Theobald	Different	99.69%
The Younger Brother	Dekker	Different	99.55%
The Younger Brother	Shakespeare	Different	99.11%

*Note*: Plays highlighted in green indicate a play’s match with a playwright’s psychological signature. Plays highlighted in light red indicate a play’s non-match with supplemental playwrights. Plays highlighted in bright red indicate a play’s non-match with Behn’s psychological signature. Unmasking data was created by averaging results across 20 randomized iterations.

The results of the unmasking procedure identified only 2 of the questioned plays as having been authored by Behn: *The Revenge* and *The Younger Brother*. All remaining questioned plays were identified as extremely poor authorial fits for Behn, as well as the supplemental authors. It is also quite promising that none of the 5 questioned plays were identified as having been written by any of the “supplemental” playwrights (Shakespeare, Fletcher, Theobald, Dekker, Marlowe, and Rowley).

## Mental profile mapping: A new authorship attribution method

Having established baseline results and expectations for the questioned plays in the current authorship space using the unmasking method, a more thorough and thoughtful consideration of the Mental Profile Mapping procedures can now be conducted. As stated earlier, a considerable drawback of most authorship attribution methods is their opacity when it comes to extracting psychological insights about a text’s author. Whereas the results of the unmasking method are fairly stark and closed to deeper interpretation, the methods of the Mental Profile Mapping approach facilitate a psychological analysis of authorship attribution results. The benefit of such an approach is quite important to authorship attribution as a field: by combining the information gain of psychological insights with statistical analyses, a more robust and accurate depiction of a text’s forensic history can be established. Additionally, a method that allows us to “peek under the hood” by means of decompositional techniques addresses several long-standing problems of methodological gatekeeping and transparency [5,27].

### Underlying concept

As a way to tackle the single-candidate authorship problem from a psychological perspective, this study introduces the concept of Mental Profile Mapping (hereafter denoted as “MPM”). The underlying concept of MPM is fairly simple: by assuming that several repeated psychological measurements are indicative of the same individual, as does any measure of personality [28], methods can be developed that facilitate the detection of outliers, or observations that fit poorly with the others. Because people show natural variation across time, such a method will want to look for outliers on not just isolated, singular psychological process (e.g., social orientation, emotional state) but instead for outliers across a whole battery of psychological measures. In other words, the MPM is primarily designed to help identify violations in the broad consistency of a person’s traits, including language-based measures of psychological processes [29]. If an observation is different along not just one psychological measure, but instead more generally, suspicion as to the origins of that observation can be reasonably raised.

As an analogy, imagine that you receive an occasional e-mail from a coworker, Margaret, perhaps once or twice a month. Over time, you begin to develop a general sense of Margaret’s personality. In her e-mails, Margaret seems to be a very positive person, keenly tuned in to the “here and now”, and often mentions her personal life (e.g., her leisure activities). While Margaret does not always say the same thing–she sometimes talks about the weather instead of her bowling league, and she seems less positive on some days relative to others–there is an overarching consistency to her personality that emerges in her communications.

One day, your supervisor tells you that she has received an e-mail from an unknown address, but she thinks that she knows who sent it. Your supervisor forwards you the text of the e-mail and asks “Does this e-mail look like it came from Margaret?”. As you read the e-mail, you might check to see if all of the criteria for Margaret’s psychological profile are met. You notice that the e-mail is generally positive and very present-focused. Additionally, there is a comment about recent leisure behaviors–the e-mail’s author states that they went to a concert this past week. Given that this e-mail appears to fall into the general constellation of Margaret’s mental universe, you may tentatively conclude that this e-mail does appear to fit Margaret’s bill for authorship.

The principal concept underlying MPM is fairly simple and parallels the example given above. In order to establish a typical psychological pattern for an individual, several psychological measures must be gathered from different time points. Just like the various qualities of Margaret’s writing (e.g., tone, time-orientation, content), each psychological measure–in this case, LIWC-based measures–will show some variation over time yet still be reflective of the underlying source. Just as you noticed consistencies in Margaret’s style, similar consistencies will emerge for an individual in their psychological processes (e.g., emotions, social processes, attentional processes). Under the assumption that all measurements were generated by the same person, outliers become suspect and merit further investigation.

In the following sections, the underlying statistical approach of the MPM will be laid out in detail. As with virtually all modern authorship attribution methods, the core of the MPM relies on statistical analysis of a person’s words. However, this approach offers several practical advantages over others. First, the underlying statistics upon which MPM works is particularly well-understood; such is not the case for many newer algorithms, as we are only beginning to understand the reasons for and boundary conditions of their efficacy [30]. Additionally, the inclusion of explicitly psychological information affords considerable information gain that is not found in other methods. For example, if a statistical distribution of words is similar to a person’s baseline texts, but the psychological profile is mismatched, we are able to retain a healthy skepticism of our own results that cannot be raised from purely distributional linguistic similarities of a text, again, facilitating a more well-rounded and accurate interpretation of the problem space.

### Mental profile mapping: Quantification and statistical methods

By pairing psychological text analysis with a multivariate distance measure, Mahalanobis distance [31,32], it is possible to statistically conduct the type of analysis described above. Ultimately, the goal is to assess the distance of several types of psychological processes from their respective centers for a given individual, then collapse all distance scores into a single metric that can be used to better understand the overarching patterns. For example, if a person’s emotional state is measured 20 times, it is possible to establish the average location, or “center”, of their emotional states. The same could then be done with the same person’s social orientation, decision-making tendencies, and so on, resulting in psychological centers for each of the different types of psychological measurements.

Unlike other distance measures, such as Euclidean distance, the Mahalanobis distance statistic is explicitly designed to handle inter-correlations among multivariate data. From a psychological perspective, the fact that Mahalanobis distance accounts for the covariance structure of the data allows for a more meaningful distance metric to be calculated relative to other distance metrics. In other words, the mathematical underpinnings of the Mahalanobis distance statistic is well-suited to handle the fact that psychological subprocesses are non-independent and may demonstrate overlap. For example, positive and negative emotions are not perfectly orthogonal [33], nor are interpersonal motives and behaviors [34]–interdependencies such as these exist across several of the LIWC psychological measures [19].

For the current MPM analysis, LIWC2015 was used to extract psychological data from all source texts mentioned in the unmasking section above. All LIWC measures were split into respective clusters of psychological processes according to their designation within the LIWC dictionary: style measures, complexity, function words, emotional processes, cognitive processes, perceptual processes, biological processes, motivational processes, temporal processes, spatial-relational processes, personal processes, and utterances (see Table 3).

**Table 3:** Psychological process compositions.

Psychological Process	LIWC2015 Variables included in Distance Calculation
1: Style	Analytic, Clout, Authentic, Tone
2: Complexity	Analytic, Sixltr
3: Function Words	i, we, you, shehe, they, ipron, article, prep, auxverb, adverb, conj, negate, interrog
4: Emotional	affect, posemo, negemo, anx, anger, sad
5: Social	social, family, friend, female, male
6: Cognitive	cogproc, insight, cause, discrep, tentat, certain, differ
7: Perceptual	percept, see, hear, feel
8: Biological	bio, body, health, sexual, ingest
9: Motivational	drives, affiliation, achieve, power, reward, risk
10: Temporal	focuspast, focuspresent, focusfuture
11: Relational	relative, motion, space, time
12: Personal	work, leisure, home, money, relig, death
13: Utterances	informal, swear, assent, nonflu

*Note*: Each process consists of several subdimensions that are factored together when calculating distance metrics.

As a point of clarification, it is important to note that the MPM method as described thus far constitutes a considerable departure, both in conceptual and practical terms, from a more traditional LIWC-based analyses of texts. A typical LIWC analysis in psychological research will focuses on isolated psychological markers that can be found in a person’s language. This is often done to understand specific differences between groups, or particular linguistic associations with other psychological constructs, such as self-focus or positive emotionality [35]. Such an approach is incredibly useful for theory-building and hypothesis-testing but is also particularly limited in scope and seldom takes into account the incredible complexity and interplay of psychological processes at the individual level. Conversely, the MPM method described thus far can be seen as an aggregation of lower-level psychometrics into a more unified view of the whole [34], which is inherently more psychological in nature than high-dimensional uses of LIWC measures for the purpose of variance-account maximization [36] or purely computational/ predictive approaches that focus on psychological phenomena [37]. Put simply, the MPM is by its very nature more all-encompassing than standard LIWC-based psychological research, yet is still firmly nested in the psychological sciences rather than being entirely computational and/or black box-esque in nature.

### Psychometrics of the mental profile map

#### Calculation of distance metrics

Psychometrics were calculated separately for each author in order to verify that results were generalizable across individuals and not idiosyncratic or unique to a single playwright. For the plays of Aphra Behn, only those plays designated as verified and legitimate Behn plays, including adaptations (i.e., those listed in Table 1), were included in the psychometric analysis. William Rowley was excluded from the psychometric analysis (and all subsequent MPM procedures) due to the fact that only 1 play by this author survives, precluding the ability to establish reliability over time.

Following the clustering of psychological processes, the center point of each psychological cluster was calculated separately for each author using a robust bootstrapping method (iteration *N* = 1000). For example, the “Complexity” cluster of psychological processes has a 2-dimensional center point for Lewis Theobald: the average of his “Analytic” scores for all of his plays is one dimension, and the average of his “Sixltr” scores for a second dimension. Similarly, the “Social” cluster has a 5-dimensional center point, and so on. Theobald’s center points for each process were uniquely derived from his own works, as were the center points for Shakespeare, Behn, and the remaining authors derived from their respective works. After calculating the center point for each individual for each psychological process, the distance of each play from the center was calculated using the Mahalanobis distance statistic. This effectively resulted in 13 separate Mahalanobis distance metrics per written work, each existing within an author’s mental profile map. Descriptive statistics for all underlying measures are presented in S2 File. Additional discussion of Mahalanobis distance as applied to these measures, is presented in Section D of S1 File.

The bootstrapped Mahalanobis distance procedure resulted in a separate “distance from center” score for each psychological process for each play. These scores were then squared and converted to 0–100 scores using chi-square estimation, resulting in a series of 13 distance measures for each play. Using this 0–100 scale, a theoretical score of 100 would reflect plays sitting perfectly at the psychological center of the map, and a score of 0 would reflect plays that fall extremely far from center. For example, if a play had a score of 90.5 for the perceptual processes measure, it would be relatively near the center of the author’s overall perceptual processes map. A low score, such as 10, would be extremely far away from an author’s perceptual processes center.

Note that the Mahalanobis metrics are, in a way, silent about directionality of deviations. Simply put, if a single distance measure (e.g., motivational processes) is extreme for a given play, the core interpretation is simply that this play is functionally *different* from the other works in this respect. The distance measure itself does not reveal, for example, that a certain play was deeply laden with (or bereft of) words related to motivational processes–the measure only indicates that a certain play appears to be askew in a generalized manner given the assumption that all plays were authored by the same individual. Instead, a score that shows great distance from the psychological center tells us that the *composition* of motivational processes for the play in question is markedly different from the other works of an author. While not inherently providing directionality information, these scores can subsequently be decomposed into meaningful interpretations, such as “While Shakespeare’s texts generally have high ‘affiliation’ words and low ‘power’ words, the pattern reverses for *Romeo and Juliet*, suggesting a radically different balance of motivational processes. Decomposition and interpretation of MPM analyses are later described.

#### Internal consistency of distance metrics

After calculating the “distance from center” scores for each psychological process for each play, for each author, Cronbach’s alpha was used to determine whether distance scores were consistent with each other. For example, if a play is far from the psychological center of Shakespeare’s collected works on cognitive processes, is the same play generally an outlier across *all* psychological processes, or are these distance metrics relatively isolated?

Cronbach’s alpha results are presented in Table 4. The internal consistency analysis did, in fact, find that the distances of each psychological process for each play formed a coherent single factor for all authors in the dataset. In other words, when a given play was further away from an author’s center along one set of psychological processes, it was generally further away from the center along all other psychological processes as well. These results were universally true and not isolated to a specific author or subset of authors.

**Table 4:** Internal consistency of distance measures for each author.

Author	Cronbach’s Alpha of Distance Measures
Behn	.66
Fletcher	.54
Marlowe	.64
Shakespeare	.52
Theobald	.70
Dekker	.66

*Note*: These results are congruent with other work on the internal consistency of psychological measures of language [19] and demonstrate that language-based measures of psychological processes do, in fact, vary in unison from the center.

The description of the MPM steps performed up to this point is, admittedly, rather intricate. As an illustrative example of the procedures described thus far, Table 5 presents descriptive statistics and inter-item correlations for all distance measures for Behn’s plays. Additionally, a simplified “step-by-step” description of the MPM process is presented in Section E in S1 File to help readers more clearly understand the steps of the process, as well as apply this method to their own work.

**Table 5:** Summary statistics and inter-item correlations for all distance measures calculated for the verified plays of Aphra Behn (i.e., excluding questioned plays).

Distance Measures	Mean (SD)	1	2	3	4	5	6	7	8	9	10	11	12
1: Style	46.24 (26.87)	–											
2: Complexity	48.05 (30.81)	-0.152	–										
3: Function Words	45.34 (14.32)	0.410	0.281	–									
4: Emotional	49.08 (28.42)	0.337	-0.234	-0.143	–								
5: Social	50.29 (28.67)	0.210	-0.200	-0.324	0.230	–							
6: Cognitive	47.14 (27.31)	0.019	0.179	0.439	-0.388	-0.317	–						
7: Perceptual	50.36 (28.47)	0.163	0.301	0.037	0.188	0.626	-0.343	–					
8: Biological	54.16 (29.81)	0.522	0.006	0.057	0.467	0.503	-0.321	0.567	–				
9: Motivational	53.49 (31.59)	0.497	-0.474	-0.141	0.618	0.363	-0.095	0.193	0.391	–			
10: Temporal	47.03 (30.58)	0.297	0.380	0.232	-0.176	-0.196	0.312	-0.242	-0.116	-0.178	–		
11: Relational	47.01 (26.66)	0.641	-0.118	0.286	0.277	0.170	-0.177	0.174	0.448	0.052	-0.071	–	
12: Personal	49.87 (29.72)	0.542	0.072	0.198	0.545	0.154	-0.269	0.194	0.714	0.276	-0.035	0.515	–
13: Utterances	50.41 (29.39)	-0.221	0.555	0.122	0.030	-0.224	0.118	0.088	0.209	-0.271	-0.016	-0.219	0.350

*Note*: The resulting Cronbach’s alpha for all distance measures (α = .66) suggests sufficient intercorrelation to constitute collapsing the distance measures into a single, meaningful psychological metric.

In effect, for each of the plays of Apha Behn, a separate Mahalanobis distance score was calculated for each of the 13 broad psychological processes captured by LIWC. Put another way, all 16 of Behn’s plays were each assigned 13 separate “distance from center” scores, one for each broad cluster of psychological processes. Across all 13 psychological processes, Behn’s plays were, on average, a modest distance away from the center points, with all scores hovering closely around 50. Furthermore, the distance scores for each of these measures were strongly intercorrelated, meaning that variation across all psychological processes were relatively harmonized with each other. For example, where Behn’s plays showed drastic deviation from (or adherence to) the center in terms of emotional processes, so too did they show considerable deviation in biological and motivational processes, and so on.

The fact that the psychological distance scores all converge on similar information facilitates 2 useful procedures in moving forward. First, given that all distance measures appear to be reflecting a similar phenomenon (distance from the “psychological center” of the map), one is able to collapse all distance scores related to a particular psychological process down to a single score. In this case, the median is preferred over the mean to prevent undue influence of a single psychological process outlier. Much like the unmasking process, drastic variations along one or two psychological processes may be superficial (e.g., different themes between works) and not reflect meaningful psychological differences. Use of the median over the mean helps to reduce excessive influence of superficial extremities. However, in this case, mean and median distance scores showed a strong correlation (*R* = +0.96) and the choice of aggregation method did not impact results. Therefore, the median of all distance scores was calculated for each play, resulting in a single 0-to-100 score that signified the strength for the case of an individual’s authorship for any given work (e.g., Behn’s *M* = 48.56, *SD* = 16.41). This final score is essentially a shorthand metric that tells us the degree to which a play is generally far away from the “psychological center” of the map across a battery of psychological processes, in which case it would merit further investigation/explanation.

Second, one can meaningfully reduce the number of dimensions from 13 psychological distance scores to 2 using multidimensional scaling (e.g., a principal components analysis). Reducing the dimensionality is primarily useful in this case to create a visualization that roughly represents how far away from the center each play rests. This allows for the consumer of these results to more easily and intuitively understand the concept of the MPM method, providing a visual reference for discussion, analysis, and interpretation of results. Given the nature of single-candidate authorship attribution problems and the inclusion of a relatively small sample of works, a mental profile map allows us to get a sense of how far away a questioned work sits from the overall psychological center. Questioned works that show relatively low MPM scores should then be subjected to further scrutiny and questioning.

**Fig 1:**
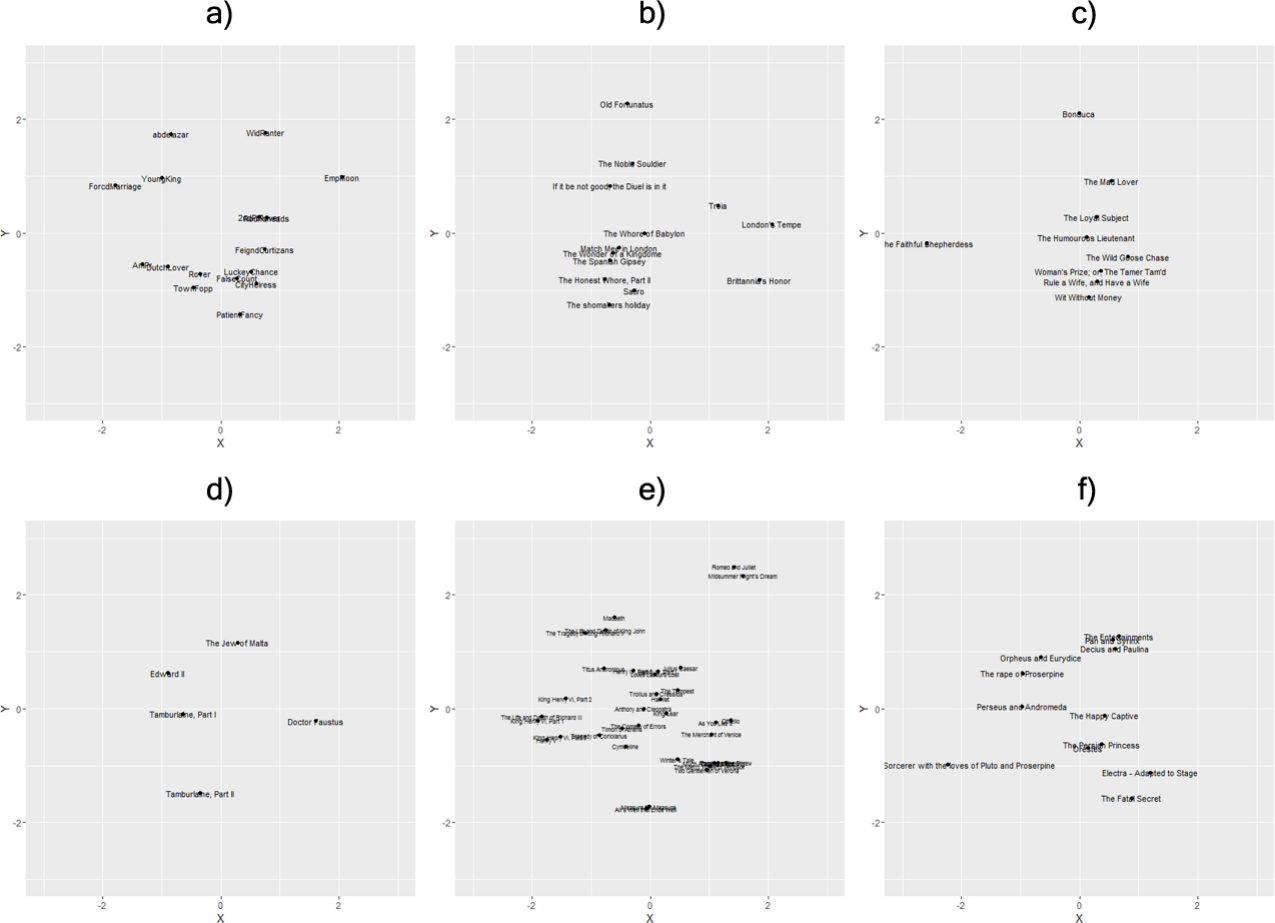
Visualized mental profile maps of 6 playwrights: a) Aphra Behn, b) Thomas Dekker, c) John Fletcher, d) Christopher Marlowe, e) William Shakespeare, and f) Lewis Theobald. *Note*: The center point (0,0) is the approximate “psychological center” for each playwright when collapsing across all 13 psychological process categories. Note that the projection of distance measures down to 2 dimensions does result in some distortion, and this graph should be interpreted as an approximation of the “true” location of each play. In other words, some plays may actually be somewhat closer to (or farther from) the center of each map than what the image may suggest.

MPM visualizations for all authors included in MPM analyses are presented in Fig 1. Note that while each author may have 1 or 2 plays that stray from the broader cluster of their psychological maps, all plots show a central “gravity” that visually represents a psychological center across all mental processes. In other words, all written works by all playwrights exhibit a tendency to radiate out from the psychological center of their respective map, demonstrating that each author’s works deviate from their own, internal norm across an entire psychological spectrum rather than single, more atomic psychological processes.

### Testing the mental profile map approach for authorship tasks

In the case of an authorship attribution task, we can adopt the MPM approach by creating a map using all of an author’s known works and, in turn, also including each work of questionable origins. By doing so, one is able to generate scores for each individual questioned work and make judgments regarding how well the questioned works fit into the larger picture. This is the approach that was adopted for all MPM analyses reported below.

As an initial test of the utility of the MPM approach in authorship attribution tasks, it is useful to first examine its performance in cases where all works are of known authorship. This can be done by performing similar procedures to those described above, albeit with some “bogus” insertions of comparable works by other known authors. It is possible, for example, to insert a play by William Shakespeare into Aphra Behn’s map. By inserting bogus plays into the MPM procedures described above, we are essentially operating under the *a priori* false pretense that Behn actually authored Shakespeare’s play. Operating under this bogus assumption, we should be able to easily spot the false insertion due to its drastic pulling away from the MPM center, both statistically as well as visually.

**Fig 2:**
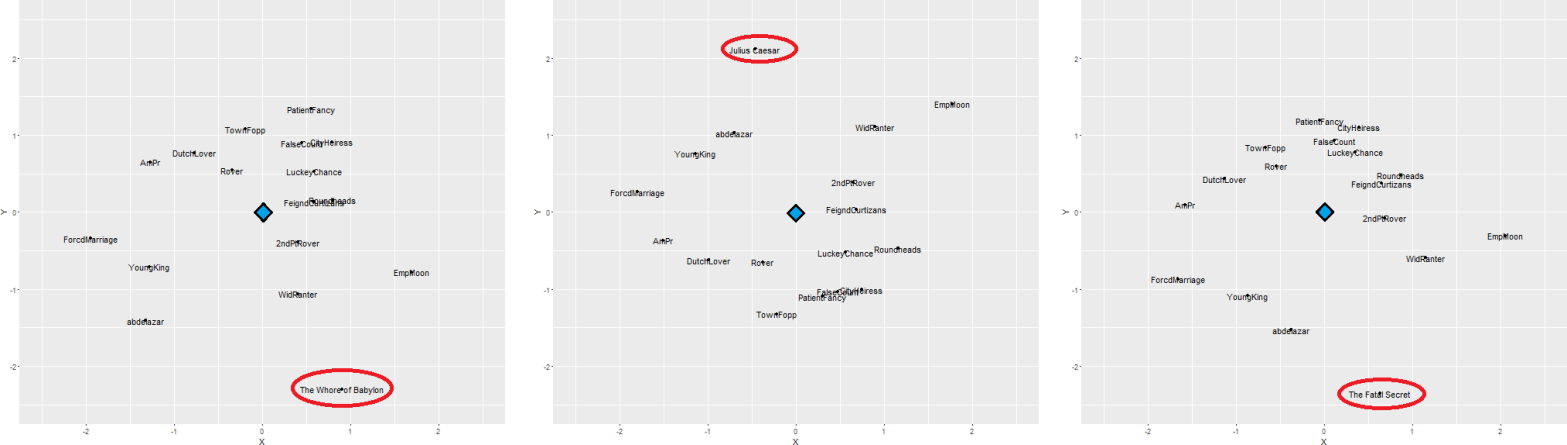
Visualized mental profile maps of Aphra Behn when including bogus plays by other playwrights. *Note*: The blue diamond denotes the psychological center of each map. Bogus plays are circled in red and stand out considerably from the rest of the map in each case. Bogus plays include Dekker’s *The Whore of Babylon* (left), Shakespeare’s *Julius Caesar* (middle), and Theobald’s *The Fatal Secret* (right).

For this test analysis, 3 plays were chosen by a random number generator from the supplemental playwright corpus. These 3 random plays included *The Whore of Babylon* by Thomas Dekker, *The Fatal Secret* by Lewis Theobald, and *Julius Caesar* by William Shakespeare. Each play was, in turn, inserted into the corpus of verified plays by Aphra Behn–the MPM procedures described in the preceding section were then performed. This resulted in 3 separate “maps”, one for each run of the MPM procedure with the inclusion of each bogus play insertion. Numeric results are presented in Table 6; visualizations are presented in Fig 2.

**Table 6:** Results of the MPM analysis.

Author	Title	Mental Profile Map Score
Behn	The Lucky Chance	76.59
Behn	Sir Patient Fancy	69.41
Behn	The Young King	68.98
Behn	The Feigned Courtesans	61.26
Behn	The False Count	60.56
Behn	The Town Fop	60.56
Behn	The Dutch Lover	58.88
Behn	The Rover, Part I	53.59
Behn	The City Heiress	50.00
Behn	The Roundheads	46.36
Behn	The Forc'd Marriage	45.76
Behn	The Rover, Part II	42.13
Behn	The Amorous Prince	41.02
Behn	Abdelazer	33.98
Behn	The Emperor of the Moon	24.43
Shakespeare	Julius Caesar	24.20
Theobald	The Fatal Secret	21.11
Dekker	The Whore of Babylon	18.09
Behn	The Widow Ranter	17.64

*Note*: MPM scores for plays marked as “Behn” authorship are averaged across each MPM analysis (correlation with Behn-only analysis: *r* = .95). Works highlighted in yellow are those that were artificially inserted as bogus Behn plays.

Results from the “bogus play” MPM analysis were extremely promising. All 3 bogus plays that were inserted are markedly distinct from the rest of Behn’s map. Numerically, all 3 insertions scored extremely low for fit in Behn’s corpus, with only one verified Behn play (*The Widow Ranter*) scoring as a worse fit than all three; the outstanding Behn play is discussed later. Visually, each bogus play was also quite distinct, falling well outside of Behn’s relatively tight ring of verified works. These results strongly suggest that this method is useful for identifying gross psychological departures from an author’s norm or, in this case, works that show a psychological signature of someone other than an author in question.

## Results: Mental profile map analysis of questioned plays

The above analyses show a strong potential for the use of MPM in authorship attribution tasks. An analysis of the questioned Aphra Behn plays was thus performed in a manner parallel to that described above. Rather than inserting bogus plays into the mental profile map, however, each questioned work was inserted in turn. Numeric results from the MPM analyses are shown in Table 7.

**Table 7:** Results of the MPM analysis. MPM scores for plays marked with “Behn” authorship are the result of the MPM analysis that included only verified Behn plays. Works highlighted in yellow are those of questioned authorship. Higher Grand MPM scores are indicative of plays with a general low distance from center (i.e., a better fit with Behn’s mental profile map).

Author	Title	Grand MPM Score (Median)	1	2	3	4	5	6	7	8	9	10	11	12	13
Behn	The Lucky Chance	72.02	69.42	96.94	68.38	14.84	50.54	72.02	75.64	75.21	3.04	94.39	81.37	62.60	87.84
Behn	The Young King	70.56	83.71	44.34	73.98	70.56	47.75	19.60	90.49	81.69	69.88	33.71	85.94	75.14	12.98
Questioned	The Younger Brother	67.60	67.60	68.81	29.27	54.76	27.29	88.03	33.52	53.14	71.21	77.16	10.11	88.00	79.82
Behn	Sir Patient Fancy	66.26	94.83	29.76	29.22	66.26	73.22	29.03	39.74	77.54	83.89	92.20	70.82	47.62	5.38
Behn	The Feigned Courtesans	62.54	41.50	60.35	36.47	93.81	62.54	21.89	48.67	65.31	85.75	84.21	30.48	84.60	66.79
Behn	The False Count	58.94	36.57	93.89	42.78	68.39	17.45	69.25	67.80	57.58	76.97	30.20	28.58	58.94	89.54
Behn	The Town Fop	56.17	56.17	72.93	65.45	23.58	50.61	75.45	39.49	76.23	54.98	45.95	28.91	72.18	88.46
Behn	The Rover, Part I	54.98	91.05	14.78	54.98	68.63	32.55	73.98	13.39	35.02	85.04	41.39	90.57	80.09	40.48
Behn	The City Heiress	50.75	50.75	49.00	36.70	68.04	82.15	18.78	96.26	68.83	75.59	38.96	13.39	45.18	77.44
Questioned	The Revenge	49.78	92.11	90.84	28.70	4.30	7.51	31.98	68.13	58.54	59.78	70.88	45.73	32.47	49.78
Behn	The Forc'd Marriage	45.99	45.99	13.37	25.63	31.94	88.31	11.08	68.95	84.46	65.84	9.33	56.16	80.95	29.76
Behn	The Roundheads	44.51	44.51	16.89	55.40	49.21	53.77	85.26	35.96	27.67	78.52	59.36	25.46	7.69	3.03
Behn	The Amorous Prince	42.31	29.00	19.26	33.14	42.31	88.81	70.89	73.44	70.77	79.00	13.71	44.97	17.98	38.26
Behn	The Rover, Part II	40.83	22.85	64.91	40.83	40.42	3.79	27.25	31.95	54.26	20.72	45.90	52.28	41.12	59.97
Behn	The Dutch Lover	40.31	32.16	31.30	39.82	98.54	60.88	29.40	40.31	81.61	29.74	12.27	69.63	87.87	78.13
Behn	Abdelazer	35.53	8.56	92.99	36.36	20.38	74.22	35.53	71.12	6.14	5.37	35.97	45.08	9.91	31.47
Questioned	The Counterfeit Bridegroom	34.60	17.31	86.96	30.93	34.60	2.51	43.92	42.09	87.44	35.15	10.21	5.04	85.49	29.15
Questioned	The Woman Turned Bully	33.58	3.61	69.86	31.66	18.59	59.72	20.98	33.58	55.48	44.41	5.25	6.53	52.57	96.00
Questioned	The Debauchee	32.34	37.53	32.34	29.25	2.46	29.24	45.32	8.71	26.37	39.47	20.70	49.54	39.25	66.22
Behn	The Widow Ranter	20.03	20.03	61.74	46.74	2.12	4.40	86.16	1.48	1.34	3.12	99.67	4.71	20.69	48.53
Behn	The Emperor of the Moon	15.23	12.70	6.35	39.41	26.21	13.65	28.59	11.05	2.89	38.38	15.23	23.70	5.30	48.47

*Note*: MPM Distance Measures Key: 1: Style, 2: Complexity, 3: Function Words, 4: Emotional, 5: Social

6: Cognitive, 7: Perceptual, 8: Biological, 9: Motivational

10: Temporal, 11: Relational, 12: Personal, 13: Utterances

**Fig 3:**
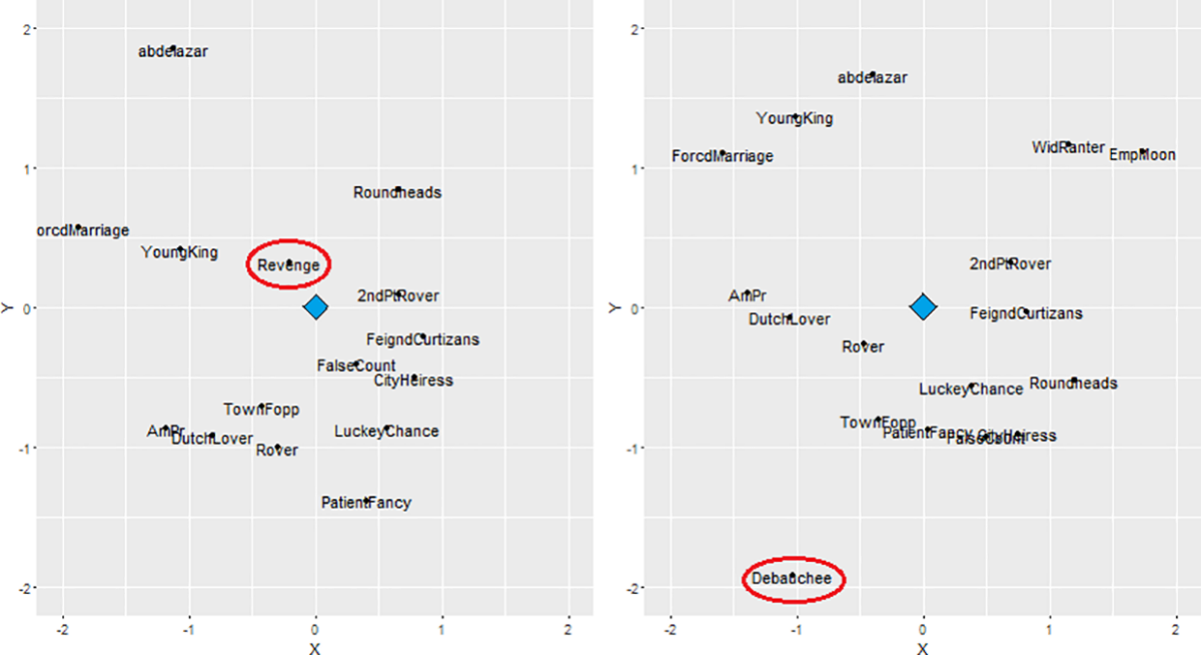
Visualization of Behn’s mental profile map when including *The Revenge* (left), a play that shows a strong MPM score, and *The Debauchee* (right), a play that shows a weak MPM score. *Note*: In both maps, the blue diamond represents the composite center of the map. As with Figs 1 and 2, the locations of each play should be considered slightly distorted approximations.

In this case, an analysis of the MPM scores would suggest that 2 questioned plays, *The Younger Brother* and *The Revenge*, show psychological patterns that are fairly typical of Behn’s corpus of plays altogether. Indeed, both of these questioned plays fare above average in their comparison to Behn’s works of verified authorship. Conversely, the remaining three questioned plays show rather low MPM scores, suggesting a moderate-to-high psychological distance from verified Behn works. The patterns are particularly striking when considering visualizations of the questioned plays’ locations within the mental profile maps (examples presented in Fig 3). When interpreting the map visualizations note that, on average, the questioned play *The Revenge* appears to be highly prototypical of Behn’s mental profile, falling close to the center of the map. Plays with rather low MPM scores, such as *The Debauchee*, show rather distant positions from the center and break from the “orbiting” placement of most other plays in terms of its psychological profile.

Taking both the MPM scores and visualizations together, these analyses suggest that 3 of the 5 questioned plays–*The Debauchee*, *The Woman Turned Bully*, and *The Counterfeit Bridegroom*–remain highly suspect regarding Behn’s authorship. These results are perfectly convergent with those provided by the earlier “unmasking” method. Given the high reliability of the language-based measures used for the current analysis, as well as the impossibility of achieving the “just right” balance between nearly 80 language dimensions without the aid of computerized systems, these results are extremely unlikely to occur by chance.

### Mental profile map decomposition

#### Decomposition of questioned plays

A primary benefit of the MPM method over more opaque machine learning methods is the ability to decompose the results into interpretable, meaningful units of analysis. Rather than receiving a hard probability score as the final result, it is possible with the MPM method to peer under the hood and look for reasons as to *why* a given text appears to be a poor fit. This can be particularly useful when looking for clues as to a work’s true author (if identified as a poor fit), or better understanding a drastic variation (e.g., a recent traumatic event or other upheavals) when authorship is certain.

The results presented above in Table 7 include each of the 13 distance metrics for each play, allowing us to manually inspect those psychological processes that appear to be driving the effects above. For all 3 questioned plays that show very low MPM scores (i.e., *The Debauchee*, *The Woman Turned Bully*, and *The Counterfeit Bridegroom*), it is clear that they generally show below-average MPM scores across all measures, suggesting a generally great distance from Behn’s psychological center along most psychological processes. Table 8 highlights those psychological processes along which the 3 low-scoring questioned plays are most discrepant (i.e., MPM scores < = 20).

**Table 8:** Decomposed categories of psychological processes that showed particularly great distance from center for the 3 questioned plays with poor support (i.e., MPM scores < = 20) for Behn’s authorship.

Play Title	Highly Discrepant Psychological Processes
The Counterfeit Bridegroom	Style, Social, Temporal, Relational
The Woman Turned Bully	Style, Emotional, Motivational, Temporal
The Debauchee	Affect, Perceptual

Once broad psychological discrepancies have been identified, it is possible to then return to the raw data to examine the specific psychological constructs that are driving the differences between the questionable plays and Behn’s overarching mental profile. In doing so, one can reference the raw scores underlying the MPM distance scores (available from the corresponding author by request) to look for extremities.

In a manual analysis, it is possible to see that *The Counterfeit Bridegroom* scores extremely high on stylistic measures such as clout and authenticity, and overall social words, family words and friend words. Additionally, this play showed extremely low past-focus and high future-focus within the dataset, and a generally high score on ‘time’ words from the relational processes cluster. This combination of extremities suggests an author with several discernible psychological characteristics: an extremely socially focused individual with strong social standing, and likely an individual who is also highly goal-directed in their day-to-day behaviors, as evidenced by the high use of future and time concepts.

Similarly, the play *The Woman Turned Bully* showed a number of extreme psychological differences from the profile extracted from Behn’s other works. In decomposing the psychological processes of this play, several major differences were apparent. *The Woman Turned Bully* exhibited extremely high authenticity scores, very low affect words (including both positive and negative emotions, and negative emotion subtypes), and low past-focus and high present-focus. Additionally, this play exhibited large differences from Behn’s profile in both reward words (very high) and risk words (very low). Taken together, the psychological profile of this play suggests an author who is extremely impulsive, focused on the “here and now”, low in self-monitoring, and possesses a strong drive for reward at the cost of risk sensitivity.

Of the 3 questioned plays, *The Debauchee* is perhaps the most generally different from Behn’s mental profile, yet in very few extreme ways. In emotional terms, for example, this play included extremely high use of general negative emotion words (but low use of specific negative emotion words, such as sadness or anger) and low use of positive emotion words. The other extremities for this play occurred in the domain of perceptual processes, with this play exhibiting extremely low use of perceptual words (e.g., “see” words and “feel” words), but high use of words related to sound. This small combination of extremities is generally difficult to interpret; the broader pattern of a more generalized distance from Behn’s profile may instead simply suggest a person whose psychology is fundamentally different from Behn in most ways.

#### Decomposition of Behn’s outlying plays

In the course of the MPM analyses, 2 additional plays that were included as accepted works by Behn also demonstrated an extreme divergence from the psychological center of Behn’s map. Like the questioned play *The Debauchee*, *The Emperor of the Moon* exhibited a broad, generalized difference from the mental profile of Behn, with no specific clusters of psychological processes appearing to be particularly outstanding (i.e., MPM score < = 20); instead, virtually all processes were outstanding. *The Widow Ranter* was highly discrepant in both the “bogus insertion” analysis described earlier as well as the MPM analysis of questioned plays. *The Widow Ranter* shows a more unique pattern: several of the psychological processes (complexity, cognitive, and temporal) appear to be a very close fit with Behn’s profile, whereas the others have varying degrees of distance between her psychological center.

Unlike the results provided by the “unmasking” method, which might only suggest that these 2 plays would be difficult to classify, we are able to look for psychological reasons as to why such plays may vary so drastically. Given the historical context, it is difficult to conclusively determine the forensic history of these 2 accepted plays. One possible explanation for the extreme positioning of these 2 plays would revolve around the nature of collaboration amongst playwrights during the time of Behn. For example, it is generally accepted that notions of authorship and collaboration were rather different than those of today, and there is extensive evidence that uncredited joint authorship was commonplace within the King’s Company and Duke’s Company [38,39], both of which were groups for which Behn worked. As such, it is possible that these plays were either only minimally authored by Behn, or perhaps heavily revised by other authors.

Additionally, both *The Widow Ranter* and *The Emperor of the Moon* were likely written near the time of Behn’s death in 1689 [18,40]. In her final years, Behn’s health was ailing and the nature of her work saw a shift, including various other types of prose and translations. The fact that Behn began to suffer from poor health may have been coupled with the accompanying psychological shifts [41] and could potentially explain the drastic change in the mental profile of these 2 plays relative to the other works of Behn. Lastly, these results may also suggest that the veracity of their authorship by Behn requires additional scholarship and research.

## Discussion

The current study used the works of several playwrights to introduce a new, sophisticated authorship attribution method for cases of single-candidate attribution tasks. Results from this new Mental Profile Mapping method were also compared with a powerful attribution method from the computational sciences known as “unmasking”. Throughout the course of the study, several goals were achieved: the development of a new method for single-candidate problems, the shedding of light on a specific authorship question, and the establishment of psychometric possibilities in the realm of high-dimensional psychological profiling.

### Mental profile mapping method

The current study demonstrated the underlying methods and utility of a new method, MPM. The MPM approach to authorship attribution possesses a number of benefits over other methodologies in the authorship attribution space. Foremost among these benefits is that it is, to the author’s knowledge, the only existing “pure” method for psychological profiling in single-candidate authorship attribution. Unlike other methods that require the introduction of additional data from other sources, either in the form of imposters or comparators for modeling, the MPM method requires “ground truth” text from only a single source. In cases where comparable texts from other authors are unavailable, this feature of the MPM method is particularly valuable.

Results from all analyses in the MPM framework were particularly strong. The psychometric assessment of the MPM as a methodology revealed that its underpinnings do, in fact, form a coherent construct that suggests language samples vary not just in single, isolated ways from a person’s psychological center but, instead, across all included language-based measures of psychology in unison. Additionally, “ground truth” tests that included known bogus insertions performed extremely well–the MPM method was able to capture false Behn plays almost perfectly.

As demonstrated in the current study, the MPM method need not exist or be performed in isolation. In cases where additional “supplemental” texts can be made available for the purposes of establishing baseline functions, several advanced machine learning methods may be used in conjunction with the MPM approach to strengthen an inquirer’s confidence in the results. For example, if multiple, radically different attribution methods converge on similar results, as occurred in the current study, increased confidence can be placed in the outcome. Additionally, features of the MPM method, such as the discrete psychological process distance scores, may be useful for inclusion as features in other authorship attribution frameworks.

Finally, a featured benefit of the MPM approach over other authorship attribution methods is that it is fundamentally a *psychological* method of authorship attribution. Insofar as psychological information can be extracted from language data, an individual performing the MPM method is able to more deeply and thoroughly examine an attribution problem by combining the results with data from other sources. By decomposing the MPM distance scores, researchers are able to identify cases of questionable origin but, also, extract a psychological profile from questioned texts. This ability may be of particular value in legal and forensic settings, where various forms of evidence and information must be considered together in order to render decisions. For example, if the MPM of an authorial suspect closely aligns with behavioral outcomes (e.g., never late to work, always polite) or personality reports from family and friends (e.g., conscientious, agreeable), a questioned work that shows radically different psychological properties (e.g., hostile and impulsive) is likely to not only show statistical differences from a candidate’s MPM, but can point to the psychological profile of the true author.

### The plays of Aphra Behn

In the current study, the MPM procedure was put to the test using the works of Aphra Behn, a prolific female playwright of the 17^th^ century. Across 2 highly distinct attribution methodologies, Behn’s unique psychological fingerprint was discernible in her work. This remained true with her original works and, additionally, Behn’s adaptations were also clearly imprinted with her unique psychological composition.

Of primary focus in the current test were 5 plays of questioned origins. The unmasking analysis and the MPM analysis converged to identify 2 of the 5 questioned plays, *The Revenge* and *The Younger Brother*, as showing a high likelihood of Behn’s authorship. The remaining 3 plays, *The Debauchee*, *The Woman Turned Bully*, and *The Counterfeit Bridegroom*, exhibited an extremely poor fit for Behn’s mental profile across both attribution methodologies. Additionally, the MPM analysis provided results suggesting likely psychological traits of the 3 questioned plays’ authors. *The Woman Turned Bully* bears the signature of a highly impulsive person with poor self-monitoring abilities. *The Counterfeit Bridegroom* exhibits language patterns that are commonly associated with individuals of particularly high social standing and a strong, goal-oriented mindset. *The Debauchee*, unlike the other 2 plays of questionable origin, did not show any particularly outstanding mental profile–rather, the embedded psychological traits appeared to be, quite simply, generally different from those of Behn.

Like all automated authorship attribution studies, care should be taken in interpreting the results of this authorship test. In the world of authorship attribution, particularly with historical data of uncertain origins, results are never a “sure thing” and must be interpreted in the light of converging evidence. In other words, the results of these analyses cannot conclusively *prove* that Behn did not have an authorial hand in *The Debauchee*, *The Woman Turned Bully*, and *The Counterfeit Bridegroom*. However, the results of both the unmasking and MPM procedures provide a strong impetus for deeper examination from domain experts researching the area of Aphra Behn’s life and work. At the very least, the 3 weak-evidence plays merit some explanation for their divergence from Behn’s verified works on the mental profile map–an explanation is further merited by the results of the unmasking method, which supports the conclusions of the MPM approach.

### Limitations

The current study does possess some limitations that should be taken into consideration. Functionally speaking, the current study includes a small sample size; only 6 to 7 authors were included for all of the analyses performed in this work. It is possible that with a greater number of authors included in the current sample, the results of all analyses may shift to favor another conclusion. Importantly, however, the unmasking method has been extensively validated and tested in previous work [20]. In other words, the unmasking method is already proven and established as a valid and powerful form of addressing authorship attribution questions. The convergence of the MPM results with the unmasking method is extremely promising. Nevertheless, future work may benefit from more extensive testing on broader samples.

Additionally, the current study was performed in a rather constrained context. In practical terms, a demonstration of the MPM method on Elizabethan, Stuart period, and Restoration era playwrights may not extend to texts of other eras or genres. Additionally, all texts used in the current analysis were of particularly healthy length. For example, in the Behn/questioned work MPM analyses, the average word count was nearly 26,000 words per play. The degree to which the MPM method would be applicable to shorter texts, such as e-mails, short letters, or social media updates, is unclear.

In spite of the constrained context, there is no reason to suspect that the procedures used within the MPM method would not extend to domains outside of the current tests. Indeed, most authorship attribution tasks are initially tested on long-form texts such as novels, yet are still viewed as applicable to other forms of language samples, assuming that all texts in a given sample are of generally comparable genres. Additionally, the language-based measures used in the current analyses have been extensively validated for the purpose of extracting psychological information from language, often including extremely short texts such as tweets [42,43].

Finally, it is important to note that MPM as it was performed in the current analysis relied heavily on the use of LIWC2015, which was developed in modern times for the explicit purpose of assessing psychological constructs from natural language samples. While the LIWC dictionary has been validated across thousands of studies and in a variety of contexts [6,7,19], the degree to which any specific LIWC—psychology link can be generalized backwards in time is not especially well-known, particularly for texts dating back multiple hundreds of years. However, there is no reason to suspect that fundamental human psychological links between language and cognition have changed substantially in such an evolutionarily brief period of time. Indeed, previous work with texts from the same era explored in this study have demonstrated that language—psychology links obtained from classical authors are not only psychometrically reliable, but conform nicely to observer reports [12]. Moreover, recent analyses of historical texts have demonstrated that psychological processes manifest in modern language can be reliably extended backwards by at least 250 years, and perhaps earlier [44–46]. Nevertheless, the particular historical nature of the current data should be weighed into any consideration of the current results.

### Future directions

Future work with the MPM method should focus on an expansion outside of the current context. The most obvious applications for the MPM method are in both legal and forensic contexts wherein a reconstruction of historical events, such as determining a document’s origins, may be absolutely vital for rendering verdicts of guilt or innocence. Further support for the conclusions of the MPM method can be provided in the context of machine learning frameworks as well; the distance metrics generated as a part of the MPM procedures will likely prove useful in the context of other machine learning and authorship attribution frameworks.

Crucially, the underlying concept of the MPM approach may possess extended value outside of forensic applications. In the modern world, technology has opened up diverse and highly complex possibilities for new assessment methods, such as the collection of rich behavioral data from smartphone technologies [47]. As interest grows in the use of rich idiographic data in the fields of mental health and medicine [48], new methods of quantifying an individual’s psychological variations over time will be required. The MPM and methods like it could, for example, be put to meaningful use in clinical settings for patients with psychotic or mood disorders. Such an approach may allow mental health providers to more accurately monitor patients’ day-to-day psychological variations and potentially facilitate faster detection of extreme, generalized psychological variations that could be diagnostic of problematic episodes [49]. Such possibilities currently remain within the scope of future research in psychology and the computational sciences.

## Conclusions

Mental Profile Mapping is an early first step in realizing the possibilities of pairing advanced statistical modeling procedures with interpretable, actionable psychological insights. Additionally, the current work highlights the future promise of better understanding the individual as a high-dimensional composite or bundle of psychological processes. As psychological and computational forensic techniques continue to advance, there will be an increasing number of opportunities to create meaningful combinations of methods from the two disciplines. Future work in the areas of psychological and computational forensics will likely benefit from increased collaboration and cross-pollination. As new techniques are needed to address increasingly complex and nuanced problems in each field, the adoption of techniques from both areas of study will help to generate more comprehensive, rigorous, and meaningful insights into the human condition.

## Supporting information

S1 File Additional information, discussions, and clarification regarding the mental profile mapping approach. (PDF)

S2 File Descriptive statistics of the underlying LIWC measures for each playwright’s corpus. (TXT)
