# Supplementary material for: Mental profile mapping: A psychological single-candidate authorship attribution method
Source: PLoS One. 2018 Jul 12;13(7):e0200588. doi: 10.1371/journal.pone.0200588 (PMC6042775; doi:10.1371/journal.pone.0200588)
Supplement: S1 File — (PDF) [file pone.0200588.s001.pdf]

Mental Profile Mapping:
A Psychological Single-Candidate Authorship Attribution Method

SUPPORTING INFORMATION

Ryan L. Boyd

Dept. of Psychology
The University of Texas at Austin

Section A: Additional discussion of authorship attribution tasks in single- versus multiple-candidate scenarios

In the world of authorship attribution, the distinction between multiple-candidate and single-candidate attribution problems can be, at times, somewhat unclear for several reasons. First, in the past decade, several methods have been developed to handle single-candidate authorship problems by translating the problem space into one of multiple-candidates [1], typically out of necessity and as a means to avoid simply detecting "novelty" via clustering or kernel methods [2], which can be overly-sensitive to superficial differences in texts. Second, the question of what constitutes a "candidate" may not conform to how we intuitively think about authorship, particularly for works where authorship is not known.

In effect, a "candidate" in authorship attribution can be thought of as, more broadly, any "entity" that authored a document. Depending on the particular research goal of a given study, a single entity may consist of anywhere from a single author to an entire publishing process, including multiple authors and subsequent editorial involvements. Essentially, then, the terms "author" and "candidate" are often used interchangeably, with the exception of cases where multiple authors are *explicitly* treated as separate candidates. Such an exception has become increasingly common as we have recently seen an increase in the number of digital collaborations wherein the *precise* contribution of each author is known, such as edits made by users to Wikia / Wikipedia [3,4]. Aside from specific studies of collaboration, however, the terms "author" and "candidate" are typically used in the literature as shorthand for the general process by which a work was created.

For example, when studying the works of Kurt Vonnegut, one must acknowledge that his writings are likely to not be *exclusively* his own. Instead, Vonnegut's writings likely include edits made by copyeditors or other individuals involved in the development/publishing process. However, the publishing machinery that helped contribute to, say, *Breakfast of Champions* is implicitly understood to be folded into the label of "Vonnegut" when conducting an attributional analyses. Similarly, while it has been generally acknowledged for some time that the works of Shakespeare largely resulted from collaborative efforts [5], these texts are generally treated/discussed as "Shakespeare" as a generic entity in authorship attribution studies, aside from the body of research that is referred to *specifically* as Shakespeare Attribution Studies [6]; in these cases, William Shakespeare as an *individual* is separated as a candidate from other potential collaborators. For attribution studies working with such classical texts, it is nearly always assumed that some degree of collaboration occurred, but that Shakespeare had the "strongest" or most dominant authorial hand in the creation of the final works that are traditionally attributed to him – hence the "Shakespeare" label.

Section B: Additional information on LIWC as a method for quantifying psychological processes in language samples

LIWC is one of the most well-established methods/systems for extracting explicitly psychological information from natural language data and is composed of 2 parts: a "dictionary" and a GUI-driven computer application. The LIWC dictionary contains word-to-category mappings for around 80 categories of words, including both common content words (e.g., emotions, social processes, biological processes) and function words (e.g., pronouns, conjunctions, articles, etc.). For example, the "cognitive processes" category contains words like "think," "understand," and "analyze," and the "articles" category contains the words "a," "an," and "the." The LIWC dictionary was itself developed using standard psychometric approaches and has been refined repeatedly over the past 20 years [7–10].

Output from LIWC is provided as a matrix comprised of filenames, text summary measures (e.g., word count, average WPS), and the percentage of words that belong to each of the dictionary's categories (e.g., % of words reflecting anxiety, % of words reflecting visual perception, % of words reflecting informal speech, and so on). These measures of each text are commonly used as would be any other psychological measure in research, such as the prediction / understanding of psychopathology, mood, social processes, and so on [11].

The relatively simple "word counting" approach that is adopted by LIWC has been found to translate remarkably well across time and research contexts, both within and outside of the social sciences. The LIWC system has been extended into fields as diverse as computer science [12,13], medicine [14], and criminal justice [15], to name just a few. Unfortunately, the literature on LIWC is far too large and diverse to cover in any reasonable amount of space (at the time of this writing, a Google Scholar search reveals over 9,500 published articles with the term "LIWC" included). It is suggested that readers who wish to dive deeper into this literature start with some relatively recent reviews, including Tausczik and Pennebaker's (2010) overview [16], Chung and Pennebaker's (2013) chapter [17], and the latest version of the LIWC psychometrics manual [10].

Section C: The assessment of authorial psychology versus "character analysis"

A common question that naturally arises when analyzing creative texts (e.g., fiction novels, dramatic plays, etc.) is the degree to which the language analyzed reflects the true psychology of the author versus the characters themselves. To reframe this idea as a question: When using psychological measures of language, to what extent are we measuring the psychological attributes of the *author* versus their ability to write realistic *characters*?

While recent years have seen some interesting attempts to measure the psychology of characters in stories (usually in the form of "personality") [18,19], such studies can be considered more of an interesting exercise in literary studies than in psychology itself. Indeed, regardless of an author's ability to write dynamic or well-formed characters, the language patterns of an author tend to permeate their texts, as do their psychological processes. For example, research has found that an author's characters have psychological language patterns that are congruent with the *author's* gender, rather than the *character's* gender [20], and other research has found that an author's language patterns in fiction novels are predictive of things such as their own longevity [21]. Such findings would be difficult to reconcile were the language of a text's characters somehow masking an author's underlying psychological patterns.

Ultimately, the explanation for such findings is that the very patterns in a person's language that are diagnostic of their psychology are not solely content-based, nor are they merely descriptive. For example, function word patterns are virtually impossible to intentionally alter, yet are diagnostic of a wide variety of phenomena, such as thinking and social styles [22,23]. Put another way, the natural language patterns that are used to assess a person's psychology are not those that are explicitly descriptive (e.g., "I am a happy person"), but are instead more generally embedded into an author's content and style [24,11]. Previous work has found this to be true even for texts that consist almost exclusively of character dialogue, such as plays [25].

Section D: Descriptive statistics and additional discussion of Mahalanobis distance for Mental Profile Mapping

Complete descriptive statistics for each LIWC measure are presented separately for each author in the accompanying Supporting Information file (S2). The reader may note that a subset of measures show moderate departure from normality, as is most clear by referencing the skew and kurtosis statistics. Note that in the current application of Mental Profile Mapping, the dataset is fairly ideal when compared to typical language analysis scenarios. Namely, in most cases, quantified language distributions tend to be skewed to a fairly high degree, showing long tails in the positive direction. Such tails tend to normalize as a text lengthens – this holds true for relatively rare words as well (i.e., hapax legomena) [26]. Given the particularly long nature of the texts analyzed for the current study, as well as the aggregation of language patterns into LIWC measures (rather than the use of n-grams or syntactical n-grams), the data in the current study did not exhibit extreme skew.

Nevertheless, and despite the low degree of skew in the current dataset relative to most language-based research, skew does still exist within the current data. Fortunately, such skew is not likely to be influential in the current context, primarily due to the robustness of the Mahalanobis distance metric to Gaussian perturbations. Recent analyses and research have found this measure to be extraordinarily robust under non-normal distributions [27], in part because of the underlying statistical methods and properties [28]. Furthermore, were the data's skew to be an undue influencer in this context, it would in fact lead to more false positives for outliers [29], thus driving down the internal consistency between measures and leading to lower Cronbach's alphas at the author-level. While such an effect would in fact strengthen the current findings (given their strong performance under potentially inflated outlier rates), it remains unlikely that the current data were influenced in such a way.

Section E: A step-by-step description of the Mental Profile Mapping procedure

In order to simplify the Mental Profile Mapping procedures for readers, as well as to facilitate its use in other work, this section outlines the basic process in a basic step-by-step manner. Below, you will find the MPM procedures presented in the order of operations performed.

1. Ensure that all input texts are comparable in terms of genre, and that all texts are authored by the same “candidate” (as described earlier in the supplementary materials).

2. Quantify all input texts using the LIWC dictionary, resulting in an output matrix of LIWC measures (columns) for each text (rows).

    a. LIWC2015 is recommended, as it is the most recent version of the software/dictionary, and includes several categories that do not exist in previous versions.

    b. Note that LIWC2015 is not required for such an analysis – older versions of the dictionary could be used in a manner that is essentially parallel to that described in the current research. Such an approach could also be extended to language categories built from topic models or word embedding clusters, however, the validity of the MPM procedures with such models remains to be explored.

3. For each cluster of LIWC variables (see Table 3 of the article), calculate a separate *n*-dimensional centroid. Bootstrapping a median is recommended over the mean.

    a. As a simplified example, separately calculate the median for the “Style” cluster categories: Analytic, Clout, Authentic, and Tone. This will give you a 4-dimensional centroid for the author’s “Style” cluster.

    b. Continuing the example, calculate the 2-dimensional centroid for the “Complexity” processes: Analytic and Sixltr.

    c. Repeat this process for the remaining 11 psychological processes.

4. For each text, calculate its Mahalanobis distance from the centroid along each of the 13 psychological processes.

    a. For example, if we have 10 texts in our authorship sample, we would calculate the Mahalanobis distance of the “Style” measures from the 4-dimenisonal “Style” centroid that was calculated in Step 3.

   b. In this example, this would be repeated for each text, for each psychological process. This would result in a total of 130 Mahalanobis Distance scores (13 Psychological Dimensions × 10 texts = 130 scores).

5. Use chi-square estimation to translate all Mahalanobis Distance scores into 0-100 probabilities. In base R, for example, the pchisq() function can be used. Note that other methods may need to be considered/introduced depending on the nature of your data [30].

6. Sanity-check results. Calculate the Cronbach's alpha for all Mahalanobis Distance scores to ensure adequate internal consistency. Following typical psychometric guidelines for construct assessment, a minimum of 0.50 is recommended. Should your Cronbach's alpha score fall below an acceptable threshold, the reliability/validity of the Mental Profile Mapping approach is more questionable.

7. Quantify the Unknown Authorship (UA) text using the same procedure described in Step 2.

8. Aggregate the UA text LIWC results into the known author's texts results from Step 2. Note that the MPM procedure should only be used on a single UA text at a time, as multiple UA texts will simultaneously exert influence across all MPM metrics that cannot be easily delineated *post hoc*.

9. Repeat Steps 3 through 5, this time including the UA text in the Mahalanobis distance calculations.

10. Calculate the median of all Mahalanobis distance scores on a text-by-text basis. This is your final MPM score for each text.

    a. The final MPM score should be interpreted as the *generalized fit* for each text within an author's corpus/canon. Should the UA text receive an inordinately low score, an explanation for its poor fit is warranted.

    b. This score, as well as the 13 separate Mahalanobis distance scores, can be inspected manually, decomposed, or even used in another supervised machine learning / authorship attribution framework.

11. Should you wish to visualize your results, all Mahalanobis distance scores can be submitted to some form of multidimensional scaling (e.g., principal components analysis). Resulting dimensions can be plotted as coordinates in 2D or 3D space. Three-dimensional plots will retain higher geometric fidelity, however, 2-dimensional plots may be easier / more intuitive for an audience to interpret. Figs S1 and S2 show the same result projected into 2D and 3D space, respectively.

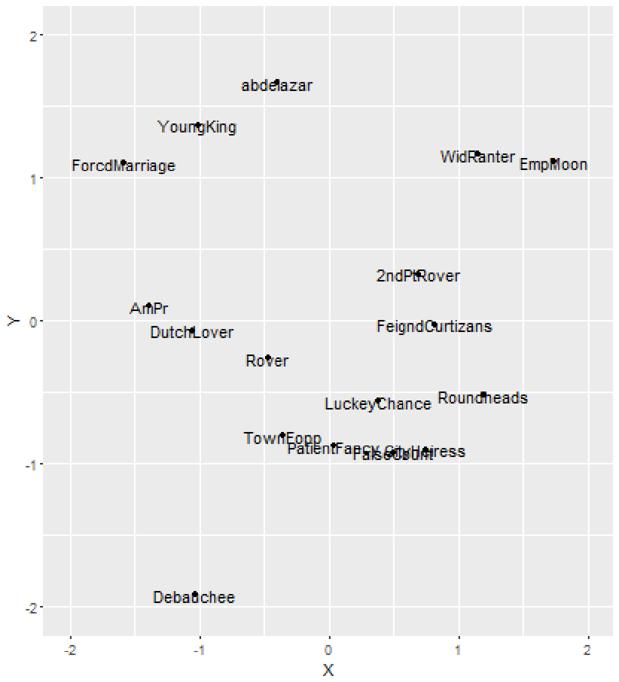

Fig S1. From the manuscript, Aphra Behn’s works projected using the MPM procedure into 2-dimensional space. In this example, *The Debauchee* was included as a test for fit.

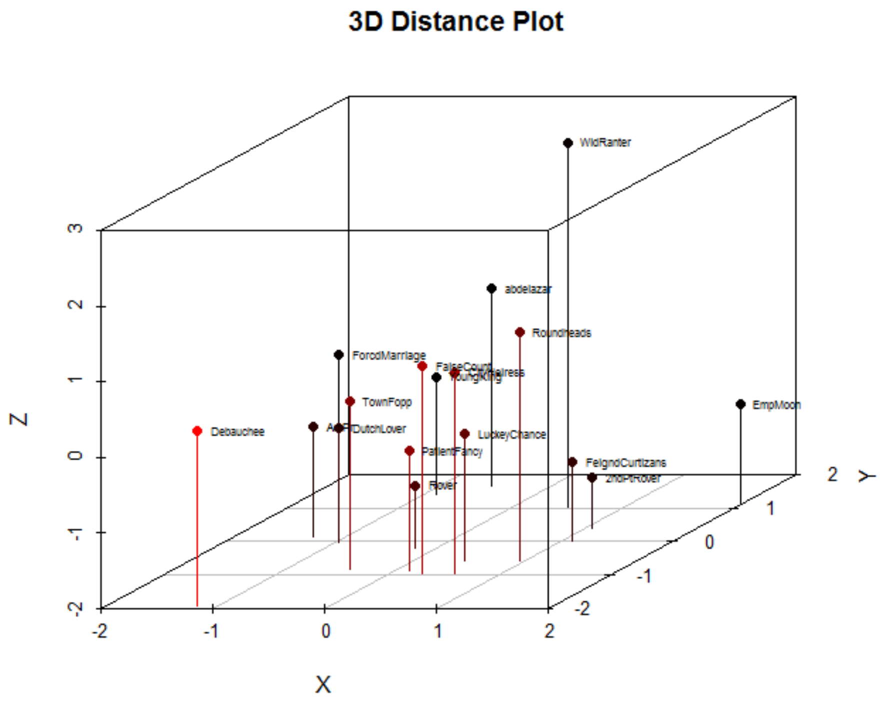

Fig S2. Identical to Fig S1, albeit projected into 3-dimensional space.

References

1. Koppel M, Winter Y. Determining if two documents are written by the same author. J Am Soc Inf Sci Technol. 2014;65: 178–187. doi:10.1002/asi.22954
2. Theodoridis S, Koutroumbas K. Chapter 1 - Introduction. In: Theodoridis S, Koutroumbas K, editors. Pattern Recognition (Fourth Edition). Fourth Edi. Boston: Academic Press; 2009. pp. 1–12. doi:https://doi.org/10.1016/B978-1-59749-272-0.50003-7
3. Dauber E, Overdorf R, Greenstadt R. Stylometric Authorship Attribution of Collaborative Documents. In: Dolev S, Lodha S, editors. Cyber Security Cryptography and Machine Learning. Cham: Springer International Publishing; 2017. pp. 115–135.
4. Macke S, Hirshman J. Deep Sentence-Level Authorship Attribution. 2015. pp. 1–7.
5. Shakespeare 1564-1616 W, Taylor 1953 G, Jowett J, Bourus T, Egan G. The new Oxford Shakespeare: the complete works . Modern cri. Oxford, United Kingdom;New York, NY; : Oxford University Press ; 2016.
6. Rudman J. Non-traditional authorship attribution studies: Ignis Fatuus or Rosetta Stone? Bull (Bibliographical Soc Aust New Zealand). Bibliographical Society of Australia and New Zealand; 2000;24: 163.
7. Pennebaker JW, Francis ME. Linguistic Inquiry and Word Count (LIWC): A computer-based text analysis program. Mahwah, NJ; 1999.
8. Pennebaker JW, Francis ME, Booth RJ. Linguistic inquiry and word count (LIWC): LIWC2001. Mahway; 2001.
9. Pennebaker JW, Chung CK, Ireland M, Gonzales A, Booth R. The development and psychometric properties of LIWC2007. Austin, TX; 2007.
10. Pennebaker JW, Boyd RL, Jordan K, Blackburn K. The development and psychometric properties of LIWC2015. Austin, TX; 2015.
11. Boyd RL. Psychological text analysis in the digital humanities. In: Hai-Jew S, editor. Data analytics in the digital humanities. New York: Springer International Publishing; 2017. pp. 161–189.
12. Choudhury M De, Gamon M. Predicting depression via social media. Proceedings of the Seventh International AAAI Conference on Weblogs and Social Media. 2013. pp. 128–137.
13. Hill J, Ford WR, Farreras IG. Real conversations with artificial intelligence: A comparison between human–human online conversations and human–chatbot conversations. Comput Human Behav. 2015;49: 245–250. doi:https://doi.org/10.1016/j.chb.2015.02.026
14. Falkenstein A, Tran B, Ludi D, Molkara A, Nguyen H, Tabuenca A, et al. Characteristics and Correlates of Word Use in Physician-Patient Communication. Ann Behav Med. 2016;50: 664–677. doi:10.1007/s12160-016-9792-x
15. Drouin M, Boyd RL, Hancock JT, James A. Linguistic analysis of chat transcripts from child predator undercover sex stings. J Forens Psychiatry Psychol. 2017;28: 437–457.

doi:10.1080/14789949.2017.1291707

16. Tausczik YR, Pennebaker JW. The Psychological Meaning of Words: LIWC and Computerized Text Analysis Methods. J Lang Soc Psychol. 2010;29: 24–54. doi:10.1177/0261927X09351676
17. Pennebaker JW, Chung CK. Counting little words in big data: The psychology of individuals, communities, culture, and history. Social Cognition and Communication. Taylor and Francis; 2013. pp. 25–42. doi:10.4324/9780203744628
18. Yuan Y, Li B, Jiao D, Zhu T. The Personality Analysis of Characters in Vernacular Novels by SC-LIWC. In: Zu Q, Hu B, editors. Human Centered Computing. Cham: Springer International Publishing; 2018. pp. 400–409.
19. Flekova L, Gurevych I. Personality Profiling of Fictional Characters using Sense-Level Links between Lexical Resources. Proceedings of the 2015 Conference on Empirical Methods in Natural Language Processing. Lisbon, Portugal: Association for Computational Linguistics; 2015. pp. 1805–1816.
20. Pennebaker JW. The Secret Life of Pronouns: What our Words Say About Us. New York: Bloomsbury; 2011. doi:10.1093/llc/fqt006
21. Penzel IB, Persich MR, Boyd RL, Robinson MD. Linguistic Evidence for the Failure Mindset as a Predictor of Life Span Longevity. Ann Behav Med. 2017;51: 348–355. doi:10.1007/s12160-016-9857-x
22. Pennebaker JW, Chung CK, Frazee J, Lavergne GM, Beaver DI. When Small Words Foretell Academic Success: The Case of College Admissions Essays. PLoS One. Public Library of Science; 2015;9: e115844.
23. Ireland ME, Henderson MD. Language style matching, engagement, and impasse in egoistic negotiations. Negot Confl Manag Res. 2014;7: 1–16.
24. Chung C, Pennebaker JW. The psychological functions of function words. In: Fiedler K, editor. Social communication. New York: Psychology Press; 2007. pp. 343–359.
25. Boyd RL, Pennebaker JW. Did Shakespeare write Double Falsehood? Identifying individuals by creating psychological signatures with text analysis. Psychol Sci. 2015;26: 570–582. doi:10.1177/0956797614566658
26. Fengxiang F, Yang Y, Yaqin W. The Probability Distribution of Textual Vocabulary in the English Language. J Quant Linguist. Routledge; 2016;23: 49–70. doi:10.1080/09296174.2015.1071149
27. Warren R, Smith RE, Cybenko AK. Use of Mahalanobis Distance for Detecting Outliers and Outlier Clusters in Markedly Non-Normal Data: A Vehicular Traffic Example. Fort Belvoir, VA; 2011.
28. Ekström J. Mahalanobis’ Distance Beyond Normal Distributions. Los Angeles, CA; 2011.
29. Tiku ML, Islam MQ, Qumsiyeh SB. Mahalanobis distance under non-normality. Statistics (Ber). Taylor & Francis; 2010;44: 275–290. doi:10.1080/02331880903043223
30. Filzmoser P. Identification of multivariate outliers: A performance study. Austrian J Stat. 2005;34: 127–138. doi:10.17713/ajs.v34i2.406
